# Lack of association between genetic variations in *CYP3A5* and blood pressure or hypertension risk in the UK biobank

**DOI:** 10.3389/fgene.2025.1490863

**Published:** 2025-05-20

**Authors:** Pia Leibold, Christelle Lteif, Julio D. Duarte

**Affiliations:** Center for Pharmacogenomics and Precision Medicine and Department of Pharmacotherapy and Translational Research, College of Pharmacy, University of Florida, Gainesville, FL, United States

**Keywords:** CYP3A5, genetic variants, genomics, hypertension, blood pressure

## Abstract

**Introduction:**

Hypertension (HTN) is a leading risk factor for several cardiovascular diseases. While some previous studies reported that *CYP3A5* variants were associated with decreased blood pressure and risk of HTN, others reported no associations. Therefore, we aimed to analyze these associations in the UK Biobank, a population large enough to have sufficient power to detect meaningful associations.

**Methods:**

The association of *CYP3A5* variants (*3, *6, *7) and CYP3A5 activity with systolic blood pressure (SBP), diastolic blood pressure (DBP), mean arterial pressure (MAP), and HTN diagnosis was analyzed in the UK Biobank (N = 487,171). Linear and logistic regression models were used, adjusting for age, sex, race, antihypertensives use, smoking status, and salt intake. Moreover, subgroup analyses were performed in Black participants, White participants, participants of East Asian and South Asian descent separately, using the same models.

**Results:**

Neither the CYP3A5 variants, nor the CYP3A5 activity showed significant associations with SBP, DBP, MAP, or HTN. In a sensitivity analysis based on different racial subgroups, only White participants showed significant associations between the CYP3A5*3 variant and slightly higher DBP (β = 0.10 mmHg, 95% CI: 0.02 to 0.18, *P* = 0.01), as well as between genotype-predicted CYP3A5 activity score and slightly lower DBP (β = −0.10 mmHg, 95% CI: −0.18 to −0.02, *P* = 0.01).

**Discussion:**

While some associations were statistically significant, the small effect sizes and lack of associations observed in the whole UK Biobank population suggest that *CYP3A5* variation likely has no impact on blood pressure related phenotypes in a general population.

## Introduction

According to the World Health Organization, cardiovascular diseases are one of the three leading causes of death worldwide (World Health Organization, 2020). The leading risk factor for cardiovascular diseases is high blood pressure through increased strain on the heart and blood vessels, which can lead to conditions such as heart attacks, strokes, and heart failure. Hypertension (HTN) contributes to the development of atherosclerosis, further increasing the risk of cardiovascular events (Whelton et al., 2018). Evidence suggests that genetic predispositions for developing elevated blood pressure exist (Garcia et al., 2003). One of the several genes implicated in HTN risk is *CYP3A5* (Xi et al., 2011). CYP3A5 is a member of the superfamily of cytochrome P450 enzymes that plays a crucial role in the metabolism of a wide variety of endogenous and exogenous substances, including drugs, steroids, and toxins. This enzyme group is particularly important as it contributes to the metabolism of many clinically used drugs such as tacrolimus, cyclosporin, and midazolam (Klyushova et al., 2022). The expression of the *CYP3A5* gene and CYP3A5 enzyme activity are highly variable among populations. While the CYP3A5*1 allele is functional, the CYP3A5*3 variant, which is most frequent in European ancestry, leads to incorrect splicing and a non-functional CYP3A5 enzyme. The CYP3A5*6 and *7 variants, which are overall rare but are relatively more frequent in African ancestry, also lead to a non-functional CYP3A5 enzyme. The CYP3A5*6 variant causes alternative splicing and deletion, while CYP3A5*7 results in a premature stop codon (Suarez-Kurtz et al., 2014).

Increased expression of *CYP3A5* in the kidney is thought to lead to elevated blood pressure, and ultimately HTN, through increased metabolism of cortisol to 6-hydroxy-cortisol, leading to increased sodium and water retention. Since the CYP3A5*3, *6, and *7 variants lead to a non-functional CYP3A5 enzyme, individuals carrying these variants may have reduced cortisol metabolism and sodium retention, potentially reducing their risk of hypertension compared to CYP3A5*1 carriers (Lamba et al., 2002; Wojnowski, 2004; Xi et al., 2011; Zhang et al., 2010). Previous studies investigating the association between *CYP3A5* polymorphisms and elevated blood pressure or HTN have shown conflicting results. Many of these studies were limited by small sample sizes, which constrained their statistical power to detect modest effect sizes (Xi et al., 2011; Galaviz- Hernández et al., 2020). The UK Biobank (UKB), with its large, well-characterized cohort and standardized recruitment and measurement protocols, provides a unique opportunity to overcome these limitations and improve the robustness of genetic association analyses. Therefore, we aimed to analyze associations between *CYP3A5* variants and blood pressure or risk of HTN development in a study cohort from the UKB to better characterize the cardiovascular relationships of *CYP3A5*.

## Materials and methods

### UK biobank study population

The study population consisted of participants from the UKB with genomic data available (Bycroft et al., 2018). All individuals were from across the United Kingdom, and those with information on age, sex, race, smoking status, salt intake, medical history, in particular, blood pressure levels and HTN diagnosis, were included. Age was defined as age at the first UKB examination, sex was based on genetic sex, race was defined as self-reported race, smoking status as self-reported smoking status, and salt intake as self-reported salt intake. Medication history included the use of antihypertensive medications, including beta-blockers, calcium channel blockers, aldosterone receptor antagonists, angiotensin receptor blockers, angiotensin converting enzyme inhibitors, thiazide diuretics, and loop diuretics (Bycroft et al., 2018). Antihypertensive drug classes were created by extracting the UKB drug code according to the drug names for the antihypertensives in the Anatomical Therapeutic Chemical classification system (KEGG BRITE database 2024; Supplementary Table S1). A binary variable was created for each of the antihypertensive classes.

### Outcomes

The association of *CYP3A5* genetic variants with systolic blood pressure (SBP), diastolic blood pressure (DBP), mean arterial blood pressure (MAP), and the presence or absence of HTN was evaluated in the entire study population. SBP and DBP were based on the blood pressure readings from the initial UKB examination. The resting blood pressure (SBP, DBP) on the left arm was measured using an Omron 705 IT electronic blood pressure monitor. After a one-minute interval, the measurement was repeated. If the automated device was unavailable, a manual sphygmomanometer was employed (UK Biobank, 2024; Supplementary Table S2). For this study, automated blood pressure readings were utilized, and if unavailable, manual measurements were used instead. Since two UKB blood pressure readings were obtained, the mean of both readings, either automated or manual, was used. MAP was calculated as MAP = DBP +1/3 (SBP–DBP) (DeMers and Wachs, 2023). HTN was defined based on ICD9 (401, 4010, 4011, 4019) and ICD10 (I10) codes for primary (essential) HTN (Bycroft et al., 2018; Supplementary Table S2).

### CYP3A5 variants

The imputed genotype data available from UKB were used in the analysis. DNA was extracted from whole blood samples from the UKB participants using the Tecan Integration Group DNA extraction system. Genotyping was performed using one of two methods: the Applied Biosystems UK BiLEVE Axiom Array by Affymetri (now part of Thermo Fisher Scientific) or the Applied Biosystems UK Biobank Axiom Array. These two systems are closely related, sharing 95% of their marker content (Bycroft et al., 2018; Welsh et al., 2017). The *CYP3A5* variants included in the analysis were the *3 (rs776746), *6 (rs10264272), and *7 (rs41303343) alleles. These variants are no ‐ function alleles and are the only polymorphisms in *CYP3A5* currently known to abolish enzyme function. A lack of or a decrease in CYP3A5 enzyme function is thought to be associated with reduced risk of high blood pressure and HTN through decreased metabolism of cortisol to 6-hydroxy-cortisol, leading to decreased sodium and water retention in the kidneys (Lamba et al., 2002; Wojnowski, 2004; Xi et al., 2011; Zhang et al., 2010). These three no ‐ function variants occur at different frequencies in various ancestral populations. Specifically, CYP3A5*3 is found in approximately 90% of people of European ancestry, while CYP3A5*6 and *7 are more common in those of African ancestry with frequencies of about 11% and 9%, respectively (Sherry et al., 2001; Supplementary Table S3).

### CYP3A5 activity score

The genotype-predicted CYP3A5 enzyme activity was calculated based on the number of tested non-functional alleles (*3, *6, or *7) present. Therefore, the presence of two non-functional alleles corresponded to an activity score of 0, one non-functional allele corresponded to an activity score of 1, and the absence of any functional alleles corresponded to an activity score of 2 (Lamba et al., 2012).

### Subgroup analysis

Given the large differences in *CYP3A5* allele frequencies by race, subgroup analyses were performed in Black participants, White participants, and in participants of South (Indian, Pakistani, Bangladeshi), and East (Chinese) Asian ancestry separately to determine whether the results of the overall population were consistent within different ancestral populations. The same single variants and the CYP3A5 activity score were tested for associations using the same models as used in the primary analysis.

### Statistical analyses

Associations of individual *CYP3A5* variants (*3, *6, *7) and genotype-estimated CYP3A5 activity with SBP, DBP, and MAP were analyzed using linear regression models, while associations with HTN diagnosis were analyzed using logistic regression models. All models were adjusted for age, sex, self-identified race, smoking status, salt intake and antihypertensive medication use (angiotensin converting enzyme inhibitors or angiotensin receptor blocker, beta-blocker, calcium channel blocker, angiotensin receptor antagonists, thiazide diuretics, and loop diuretics). Individual variant associations were evaluated assuming an additive model (A/A vs. A/a vs. a/a). The same statistical tests were performed in the subgroup analyses by race. Confidence intervals (CI) and odds ratios (OR) were reported. *P* < 0.05 was considered statistically significant. All analyses were performed in R (version 4.3.3).

## Results

### Participant population

The number of participants with phenotype and genotype data available in the UKB dataset totaled 487,171 participants (Table 1). The mean age was 56.5 (±8.09) years and 54.24% were female (Table 1). The majority of the population self-identified as White (94.24%) followed by Asian (1.93%), and then Black (1.57%; Table 1), with other racial groups each comprising less than 1% of the total population (Table 1). The most common antihypertensive medication was angiotensin converting enzyme inhibitors, followed by calcium channel blockers, beta-blockers, and thiazide diuretics (Table 1). Most participants never smoked (54.47%) and the majority never or rarely added salt to their food (55.39%; Table 1).

**TABLE 1:** Included UKB participant characteristics.

Population characteristics	Overall (N = 487,171)	Poor metabolizers (CYP3A5 activity = 0; N = 413,666)	Intermediate metabolizers (CYP3A5 activity = 1; N = 67,878)	Normal metabolizers (CYP3A5 activity = 2; N = 5,546)
Age	56.5 ± 8.1	56.6 ± 8.1	56.1 ± 8.2	53.9 ± 8.4
Sex				
Female	264,230 (54.24)	224,220 (54.20)	36,872 (54.32)	3,099 (55.88)
Male	222,941 (45.76)	189,446 (45.80)	31,006 (45.68)	2,447 (44.12)
Self-reported race				
White	459,116 (94.24)	399,653 (96.61)	57,347 (84.49)	2,038 (36.75)
Black	7,641 (1.57)	1,769 (0.43)	3,792 (5.59)	2,080 (37.51)
East Asian	1,502 (0.31)	783 (0.19)	595 (0.88)	123 (2.22)
South Asian	9,414 (1.93)	5,336 (1.29)	3,368 (4.96)	708 (12.77)
Mixed	2,839 (0.58)	1,729 (0.42)	998 (1.47)	112 (2.02)
Other	4,351 (0.89)	2,664 (0.64)	1,298 (1.91)	389 (7.01)
Smoking status				
Never	265,335 (54.47)	223,414 (54.01)	38,263 (56.37)	3,616 (65.20)
Previous	168,282 (34.52)	144,930 (35.04)	22,077 (32.53)	1,244 (22.43)
Current	51,195 (10.51)	1,863 (10.51)	7,123 (10.49)	605 (10.91)
Salt intake				
Never/Rarely	269,884 (55.39)	230,305 (55.67)	36,849 (54.29)	2,646 (47.71)
Sometimes	136,391 (28.00)	115,415 (27.90)	19,242 (28.35)	1,709 (30.82)
Usually	56,612 (11.62)	48,042 (11.61)	7,930 (11.68)	634 (11.43)
Always	23,610 (4.85)	19,411 (4.69)	3,687 (5.43)	506 (9.12)
SBP	137.9 ± 18.6	137.9 ± 18.6	137.6 ± 18.8	137.3 ± 18.9
DBP	82.3 ± 10.1	82.2 ± 10.1	82.3 ± 10.2	83.2 ± 10.5
MAP	100.8 ± 12.0	100.8 ± 11.9	100.8 ± 12.1	101.2 ± 12.3
HTN	151,091 (31.01)	123,679 (29.90)	20,558 (30.29)	1,915 (34.53)
ACEIs	52,466 (10.77)	44,835 (10.84)	7,059 (10.40)	564 (10.17)
ARBs	22,756 (4.67)	19,201 (4.64)	3,199 (4.71)	350 (6.31)
CCBs	40,517 (8.32)	33,938 (8.20)	5,854 (8.62)	714 (12.87)
BBs	36,874 (7.57)	31,565 (7.63)	4,922 (7.25)	384 (6.92)
Thiazide diuretics	36,413 (7.47)	30,803 (7.45)	5,075 (7.48)	526 (9.48)
Loop diuretics	5,753 (1.18)	4,920 (1.19)	778 (1.15)	55 (0.99)
ARAs	1,162 (0.24)	1,008 (0.25)	140 (0.21)	14 (0.25)

Values are expressed as mean ± SD or N (%) relative to the respective study population based on CYP3A5 phenotype. ACEIs, angiotensin converting enzyme inhibitors; ARAs, aldosterone receptor antagonists; ARBs, angiotensin receptor blockers; BBs, beta-blockers; CCBs, calcium channel blockers; DBP, diastolic blood pressure; HTN, hypertension; MAP, mean arterial blood pressure; SBP, systolic blood pressure.

CYP3A5*3 was the most common variant in the population with a frequency of 91%, followed by *6 and *7 at 0.3% and 0.2%, respectively. These frequencies were in concordance with those recorded in the literature in populations of European ancestry (Supplementary Table S3). The *6/*6 and *7/*7 genotypes were each present in less than 0.1% of the total study population (Supplementary Table S4). Given the large number of White participants, the majority of the overall population were poor metabolizers (Table 1).

### Associations of CYP3A5 variants with DBP, SBP, and HTN

**FIGURE 1:**
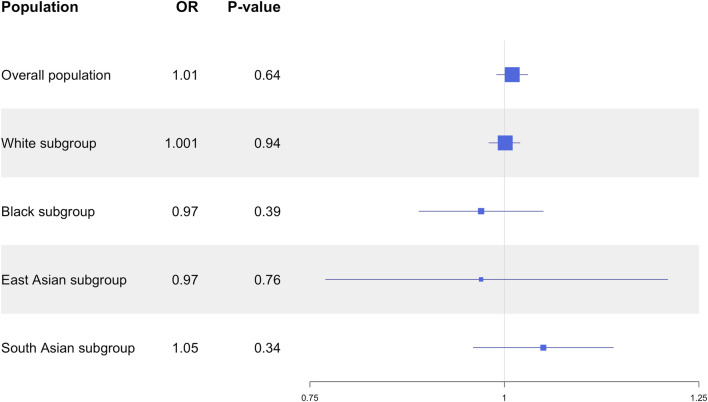
Estimated effects (β coefficients) of *CYP3A5* variant alleles on blood pressure. β coefficients were generated using linear regression models and are displayed relative to the functional CYP3A5*1 allele. The squares represent the β coefficient/effect of the variants on the outcomes with the size of the squares reflecting the precision. The lines represent the confidence intervals of each association. BP, blood pressure; DBP, diastolic blood pressure; MAP, mean arterial blood pressure; SBP, systolic blood pressure.

**FIGURE 2:**
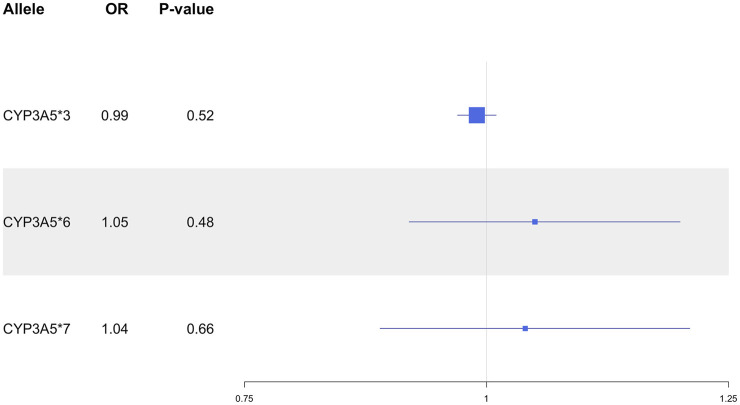
Estimated effects (ORs) of *CYP3A5* variant alleles on HTN risk. ORs were generated using logistic regression models and are displayed relative to the functional CYP3A5*1 allele. The squares represent the ORs/effect of the variants on the outcomes with the size of the squares reflecting the precision. The lines represent the confidence intervals of each association. HTN, hypertension; OR, odds ratio.

In the overall UKB population, the CYP3A5*3 allele was not associated with SBP (*P* = 0.26), DBP (*P* = 0.70) or MAP (*P* = 0.73; Figure 1), nor did it show an association with HTN risk (*P* = 0.52; Figure 2). CYP3A5*6 did not show associations with SBP (*P* = 0.53), DBP (*P* = 0.76), MAP (*P* = 0.63; Figure 1), or HTN (*P* = 0.48; Figure 2), and there were no associations found between *7 and SBP (*P* = 0.54), DBP (*P* = 0.79), MAP (*P* = 0.65; Figure 1), or HTN (*P* = 0.66; Figure 2).

### Associations of CYP3A5 activity score with DBP, SBP, and HTN

**FIGURE 3:**
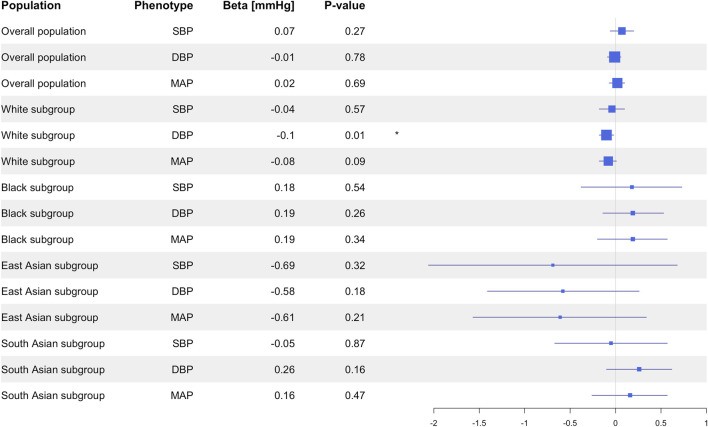
Estimated effects (β coefficients) of genotype-predicted CYP3A5 activity on blood pressure in the primary analysis in the overall population and in the different racial subgroups. β coefficients were generated using linear regression models and are displayed relative to the functional CYP3A5*1 allele. The squares represent the β coefficient/effect of the variants on the outcomes with the size of the squares reflecting the precision. The lines represent the confidence intervals of each association. BP, blood pressure; DBP, diastolic blood pressure; MAP, mean arterial blood pressure; SBP, systolic blood pressure.

**FIGURE 4:**
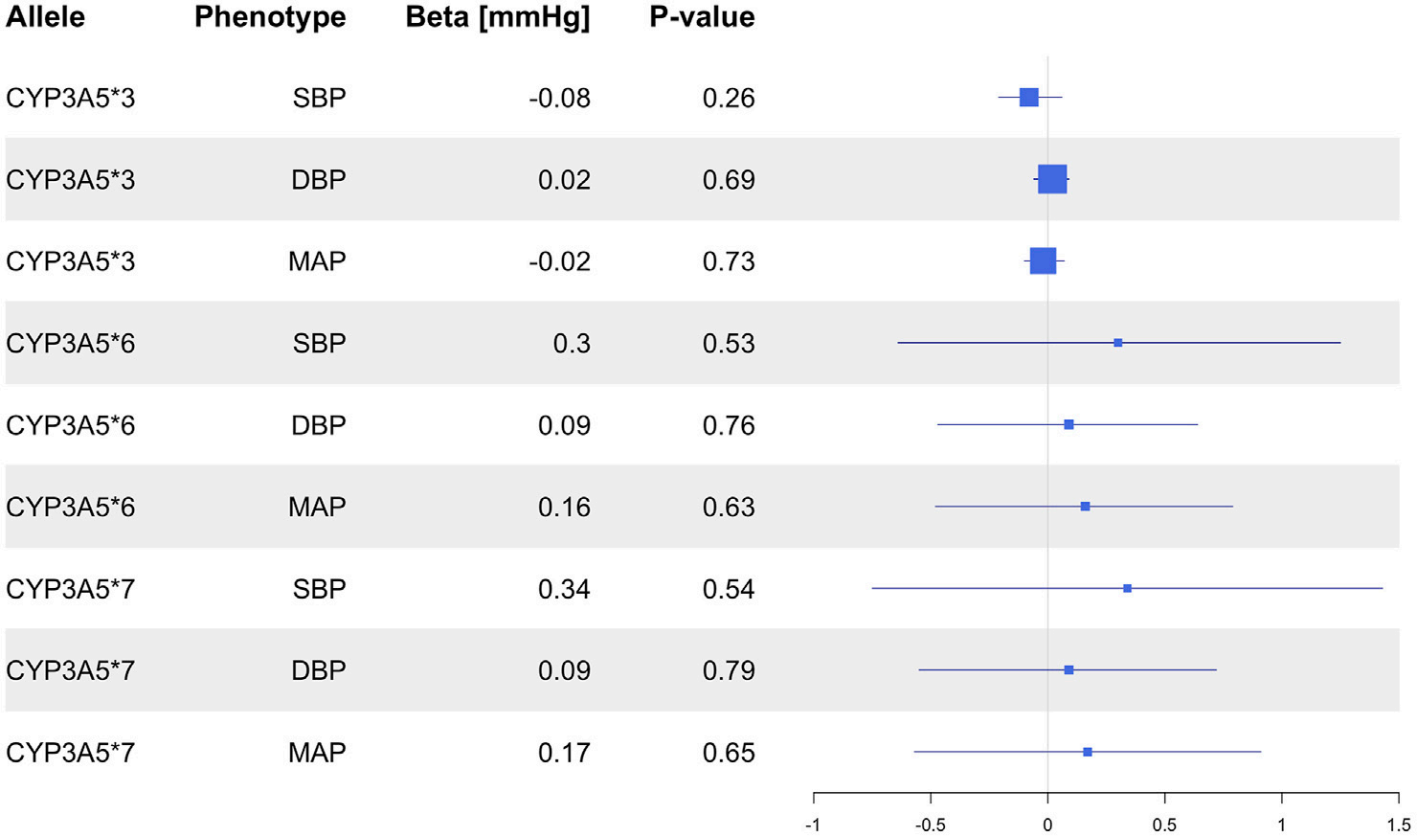
Estimated effects (ORs) of genotype predicted CYP3A5 activity on HTN risk in the primary analysis in the overall population and in the different racial subgroups. ORs were generated using logistic regression. The squares represent the ORs/effect of the variants on the outcomes with the size of the squares reflecting the precision. The lines represent the confidence intervals of each association. HTN, hypertension; OR, odds ratio.

Genotype-estimated CYP3A5 activity score was not associated with SBP (*P* = 0.27), DBP (*P* = 0.78), MAP (*P* = 0.69; Figure 3), or HTN (*P* = 0.64; Figure 4).

### Subgroup analyses

Based on the large differences in *CYP3A5* allele frequencies in different ancestral populations, a sensitivity analysis was conducted in different racial groups. The UKB study cohort was divided based on self-reported race, resulting in a subgroup of 459,116 White participants, a subgroup of 7,641 Black participants, a subgroup of 1,502 East Asian individuals, and a subgroup of 7,626 South Asian individuals.

In the subgroup of White participants, CYP3A5*3 was associated with higher DBP (β = 0.10 mmHg, 95% CI: 0.02 to 0.18, *P* = 0.01), but there were no significant associations between CYP3A5*3 and SBP (*P* = 0.54), MAP (*P* = 0.09), or HTN prevalence (*P* = 0.97; Supplementary Table S5). Likewise, CYP3A5*6 did not exhibit any significant associations with SBP (*P* = 0.77), DBP (*P* = 0.30), MAP (*P* = 0.46), or HTN (*P* = 0.30; Supplementary Table S5). Insufficient numbers of CYP3A5*7 carriers were available in this subgroup to conduct statistical analysis (Supplementary Table S4). CYP3A5 activity score was nominally associated with lower DBP (β = −0.10 mmHg, 95% CI: −0.18 to −0.02, *P* = 0.01; Figure 3), but not with SBP (*P* = 0.57), MAP (*P* = 0.09; Figure 3), or HTN (*P* = 0.94; Figure 4).

In the subgroup of Black participants, there were no significant associations between any *CYP3A5* non-functional alleles and SBP DBP, MAP, or HTN (Supplementary Table S5). There were also no associations observed with CYP3A5 activity score (Figure 3). In the East Asian and South Asian subgroups, neither CYP3A5*3 nor CYP3A5 activity score were associated with SBP, DBP, or HTN (Supplementary Table S5; Figures 3, 4). There were no CYP3A5*6, or *7 carriers in the two subgroups (Supplementary Table S4), so a statistical analysis was also not possible.

## Discussion

In this study, we aimed to assess potential associations between *CYP3A5* variants, CYP3A5 activity and multiple blood pressure-related phenotypes in the UKB population. *CYP3A5* is an important pharmacogene involved in the metabolism of several drugs. Our findings suggest that there is no meaningful association between *CYP3A5* polymorphism and blood pressure and HTN risk. While we observed a statistically significant association between CYP3A5*3 and higher DBP and CYP3A5 activity and lower DBP in the White participants subgroup, the very small effect sizes (approximately 0.1 mmHg) suggest little clinical relevance. Furthermore, the absence of significant findings in non-White subgroups reduces the significance of these associations, suggesting that the associations in White participants may reflect other influences like population specific factors instead of a general genetic association.

Prior research on the relationship between CYP3A5 variants and blood pressure has produced mixed results. Some studies have linked the loss-of-function variant CYP3A5*3 with lower blood pressure (Bochud et al., 2006; Givens et al., 2003; Jin et al., 2007) and lower HTN risk (Ho et al., 2005; Kivistö et al., 2005) suggesting that, compared to a functional CYP3A5*1 allele, the loss-of-function variants lead to lower blood pressure. Conversely, other studies found the CYP3A5*3 allele to be associated with higher blood pressure (Galaviz-Hernández et al., 2020; Fromm et al., 2005; Ho et al., 2005; Kreutz et al., 2005; Zhang et al., 2010) and increased HTN risk (Galaviz-Hernández et al., 2020). The effect sizes observed in previous studies were variable, but most of them were small (Xi et al., 2011). A meta-analysis by Xi et al. found that overall, there is no significant association between *CYP3A5* variants and blood pressure, or HTN based on the previous literature (Xi et al., 2011).

One potential reason for the conflicting findings in earlier research is that some of these studies were underpowered to detect such small effect sizes, with sample sizes of less than 500 individuals (Bochud et al., 2006; Fromm et al., 2005; Ho et al., 2005; Galaviz-Hernández et al., 2020; Givens et al., 2003; Jin et al., 2007; Kivistö et al., 2005; Zhang et al., 2010). Moreover, most studies did not account for all relevant non-functional variants, such as CYP3A5*6 and *7, which are common in some of the ancestral populations that they studied (e.g., African and Native American), potentially introducing confounding by misclassifying variant carriers as non-carriers (Galaviz-Hernández et al., 2020; Givens et al., 2003; Fromm et al., 2005; Ho et al., 2005). Notably, two studies that did consider CYP3A5 activity scores by incorporating two loss of function variants, CYP3A5*3 and *6, found no association with blood pressure rates (Fisher et al., 2016; Langaee et al., 2007). Consistent with this, our analysis of genotype-estimated CYP3A5 activity score was conducted with consideration of the *3, *6, and *7 alleles and similarly showed no clinically appreciable association with blood pressure or HTN. By including all three major functional variants, this approach offers a more comprehensive assessment, as it accounts for the participant’s haplotype profile and consequently reflects their CYP3A5 activity more accurately.

In subgroup analyses, we observed statistically significant associations between CYP3A5*3 and higher DBP as well as between CYP3A5 activity and lower DBP in White individuals (by far the largest in our population). The Black, South Asian and East Asian subgroups showed no significant associations. However, the very small effect sizes observed in the White participants subgroup suggest that each CYP3A5*3 allele is associated with only 0.1 mmHg increase in blood pressure. Likewise, each one-point increase in CYP3A5 activity score was associated with only a 0.1 mmHg decrease in blood pressure. These results underscore that statistical significance in large-scale studies does not inherently translate to clinical relevance, a critical consideration for interpreting pharmacogenomic findings. Therefore, even though these findings were statistically significant (likely only due to our large power to detect very small effect sizes), the clinical significance of these findings seems negligible, as supported by the lack of association with HTN risk. Moreover, in our study, CYP3A5*6 or *7 alone were not associated with blood pressure or HTN, which contrasts the theory that loss-of-function variants result in lower blood pressure. The results from the White participants subgroup could not be replicated in any other racial subgroup, which diminishes the generalizability and significance of the findings within the White population. Considering that our primary results did not show any associations between *CYP3A5* variants or CYP3A5 activity and blood pressure-related phenotypes and given that the significant findings in the subgroups are restricted to the White population, this suggests the presence of other factors influencing blood pressure and HTN, which may account for the differences observed between White participants and other ancestral populations, as well as the lack of findings within the whole population. These factors, rather than *CYP3A5* polymorphism, could potentially explain the significant findings in the White population.

Our study has several strengths. First, the large sample size of nearly half a million participants provides substantial power to detect subtle genetic effects if they exist. Additionally, this study included all known common genetic variants associated with loss of CYP3A5 function. This inclusive approach enables a nuanced assessment of genetic variability and its influence on blood pressure and HTN. Moreover, we adjusted for clinical factors and medications that influence blood pressure and we also conducted a subgroup analysis by race, which is particularly important given the different frequencies of *CYP3A5* variants across populations.

Our study also has some limitations. The drug usage data was self-reported, which could introduce inaccuracies, and the diagnosis codes for HTN may not be the most reliable method for determining true HTN status. Moreover, while our study included a large overall sample, the smaller sizes of some racial subgroups limit the ability to detect small effect sizes within those populations. Considering that the cohort sizes still exceeded 1,500 individuals in each subgroup, the sample sizes remain substantial enough to detect clinically significant associations. Despite including over 1,500 non-White participants, we did not observe significant associations in these subgroups. This may suggest that the associations observed in White participants might be due to environmental factors rather than a generalizable genetic effect or, given the very small observed effect size, simply chance findings. Furthermore, differences in allele frequency and limited statistical power to detect such a small effect size in non-White groups may have contributed to the lack of replication. Future studies with larger and more diverse cohorts are needed to clarify these associations. Additionally, epigenetic modifications and environmental factors may play a role in gene regulation and hypertension risk. Future research integrating more refined environmental measures may provide additional insights into gene-environment interactions in hypertension.

In conclusion, our study provides evidence of a lack of associations between *CYP3A5* variants with known functional consequences on enzyme activity, or the CYP3A5 activity score, and blood pressure and HTN. These findings suggest that common functional polymorphisms in *CYP3A5* may not play a significant role in blood pressure regulation or the risk of developing HTN. Future research should continue to explore other factors that may influence blood pressure and HTN, including potential interactions between genetic variants and environmental or lifestyle factors.

## Data Availability

The data analyzed in this study was obtained from the UK Biobank, a large-scale biomedical database and research resource. This research has been conducted using the UK Biobank Resource under application number 97332. The following licenses/restrictions apply: researchers must register, submit a research application that is approved by UK Biobank’s Access Management Team, and complete a Material Transfer Agreement. Requests to access these datasets should be directed to the UK Biobank Access Management Team, access@ukbiobank.ac.uk, through the UK Biobank Access Management System (https://ams.ukbiobank.ac.uk/ams/).
