# Supplementary material for: Lack of association between genetic variations in CYP3A5 and blood pressure or hypertension risk in the UK biobank
Source: Front Genet. 2025 May 20;16:1490863. doi: 10.3389/fgene.2025.1490863 (PMC12129758; doi:10.3389/fgene.2025.1490863)
Supplement: Supplementary file 1 [file DataSheet1.pdf]

Supplementary material

Supplementary Table S1: UK Biobank antihypertensive medication code.

Supplementary Table S2: Population characteristics, UK Biobank Field ID and description.

Supplementary Table S3: *CYP3A5* variant frequency in UKB population compared to dbSNP.

Supplementary Table S4: Genotype distribution in the study population and the different racial subgroups; frequency and percentages (%) of the CYP3A5 variant alleles in the total population and in different racial subgroups in the UKB population.

Supplementary Table S5: Results of the subgroup analysis based on self-reported race; linear and logistic regression analysis of the *CYP3A5* variants and blood pressure rates and HTN.

Supplementary Table S1: UK Biobank antihypertensive medication code.

Data Field 20003	
Antihypertensives Codes	
CCBs	1140860426,1140860358,1140861090,1140881702,1140923572,1140879802, 1141200400,1140861110,1140860356,1141187094,1140916930,1141173766, 1140861106,1140861176,1140927934,1141199858,1140861120,1141166752, 1141188836,1140888646,1141165470,1141188576,1141188152,1141145870, 1141152600,1140861190,1141188920,1141187962,1140861276,1141153026, 1140861282,1141200782,1140879810,1140861088,1141157140,1141150538, 1140911088,1140861114,1141169730,1140872568,1140926966,1140872472, 1140928226,1141162546,1140868036,1141201814,1140928212,1140861194, 1141150500,1140928234,1140927940,1140926188,1141190548,1140851790, 1141190160,1141153032,1140866546,1140866554,1141169096,1140866484, 1140866460,1141187056,1140866466,1141153316,1141153328,1140881692, 1141187774,1140888510,1141150926,1141169710,1141184390,1140861138, 1140926780,1140861136,1140917428,1141175224,1140861130,1141153454, 1140851730,1141157136,1140879806,1140926778,1140861166,1141185444, 1141180238,1140923618,1141156656,1140911698,1140861128,1141151474, 1141174684,1141167832,1141171804,1141153394,1141153400,1140861202, 1141165476,1140851784
ACEIs	1140860696,1140860706,1140860714,1140860728,1140860802,1140860806, 1140860878,11408608821140881706,1140881712,1140881714,1140888552, 1141164154,1141165470,1141165476,1141180592,1141150328,1141167758, 1141151382,1140860736,1140860750,1140860892,1140888556,1141180598, 1141200698,1140860758,1140860904,1140888560,1141188408,1140860918, 1140860764,1140860912,1140923712,1141199940,1140923718,1140860776, 1140864618,1141150560,1140860738,1141153316,1140860784,1140864910, 1141153328,1141170870,1140860790,1140864952,1141164148,1140860752

BBs	1140851484,1140851556,1140860192,1140860194,1140860220,1140860222, 1140860250,1140860266,1140860274,1140860278,1140860292,1140860294, 1140860304,1140860308,1140860312,1140860324,1140860328,1140860332, 1140860336,1140860340,1140860356,1140860358,1140860362,1140860398, 1140860404,1140860418,1140860426,1140860434,1140860492,1140860498, 1140864950,1140866704,1140866712,1140866724,1140866726,1140866738, 1140866756,1140866758,1140866764,1140866766,1140866782,1140866800, 1140866802,1140866804,1140879758,1140879760,1140879762,1140879818, 1140879824,1140879830,1140879842,1140879854,1140879866,1140909368, 1140910614,1140916868,1140922930,1140923336,1141146124,1141146126, 1141146128,1141152076,1141156754,1141164276,1141164280,1141168498, 1141171152,1141180778,1141182904,1141182968,1141187048,1141194804, 1141194808,1141194810,1140860422,1140860212,1140860180,1140860348, 1140864410,1140860172,1140866784,1140851492,1140866778,1141184324, 1140860386,1140866798,1140860244,1140851576,1140917076,1140860402, 1140875808,1140851522,1140860316,1140860334,1140860230,1140879834, 1140860320,1140860322,1141156808,1140916730,1140851480,1141172742, 1140860342,1140860338,1141187780,1140881722,1140860318
ARBs	1141151016,1141172682,1141151018,1141172686,1141152998,1141179974, 1141153006,1141187788,1141156836,1141187790,1141156846,1141193282, 1140916356,1140916362,1141145660,1141145668,1141166006,1141171336, 1141171344,1141172492,1141193346,1141201038,1141201040
ARAs	1141201244,1141201250,1140866412,1140866396,1140923282,1140851418, 1140866236,1140866244,1140866312,1140866318,1140851420,1140851508, 1140866308,1140866306
Thiazide diuretics	1140866354,1141187790,1141151018,1141172686,1141201040,1140851430, 1140851432,1140866360,1140866324,1140866328,1140860784,1140860736, 1140866162,1140860332,1140926778,1141151016,1140860404,1140860422, 1140860386,1140860562,1140860738,1140860764,1140860790,1140864950, 1140864952,1141172682,1141187788,1141201038,1140851362,1140851660, 1140866164,1140866168,1140864176,1141194794,1141194800,1140866440, 1140851332,1141194804,1141194808,1141194810,1140866136,1140866138, 1140860348,1140866446,1140866128,1140866122,1140860312,1140860316, 1140860318,1140860340,1140860342,1140860418,1140866450,1140910442, 1141146126,1140866132,1140888918,1140851336,1140866090,1140851338, 1140909706,1141180772,1141180778,1140860308,1140923336,1140864202, 1140866144,1140851364,1140866330,1140866410,1141146124,1140923276, 1141146128,1140851436,1140860336,1140866420,1140866416,1140866156, 1140866352,1140851368,1140866158,1140866422,1140923282,1140866396, 1140866402,1140866078,1141180592,1140888922,1141146378,1140917068, 1140866108,1140866110,1140866092,1140866094,1140866096,1140866102, 1140866104,1140860334,1140860322,1140860338,1140866072,1141194800, 1140864618,1140923272,1140881714,1140860398,1140864910,1140860324, 1140860328
High Ceiling diuretics	1140866426,1140866280,1140866448,1141168964,1140866282,1140866438, 1140866356,1140909708,1141195254,1141195258,1140851342,1140851400, 1140851412,1140866182,1140866192,1140866194,1141167108,1140851414, 1140866406,1140866408,1140866412,1140866418,1141169088,1140866116, 1140860320,1140866332,1140866334,1140866248,1140866262,1140888496, 1140864874,1140866200,1140866202,1140866206,1141157184,1140866210,

	1140866212,1140866084,1140866086,1140923402
ACEIs, angiotensin converting enzyme inhibitors; ARAs, aldosterone receptor antagonists; ARBs, angiotensin receptor blockers; BBs, beta-blockers; CCBs, calcium channel blockers	

Supplementary Table S2: Population characteristics, UK Biobank Field ID and description.

Patient Demographics	UKB Field ID	UKB Field Description
Sex	22001	Genetic sex
Age	21022	Age at recruitment
Self-reported race	21000	Ethnic background
Smoking status	20116	Smoking status
Salt intake	1478	Salt added to food
ACEIs	20003	Treatment/medication code
ARBs	20003	Treatment/medication code
CCBs	20003	Treatment/medication code
BBs	20003	Treatment/medication code
Use of thiazide diuretics	20003	Treatment/medication code
Use of loop diuretics	20003	Treatment/medication code
Use of aldosterone-receptor-antagonists	20003	Treatment/medication code
SBP	4080; 93	Systolic blood pressure automated reading; manual reading
DBP	4079; 94	Diastolic blood pressure automated reading; manual reading
MAP	*Self-calculated based on SBP and DBP*	
HTN	41270; 41271	Diagnoses-ICD10; Diagnoses-ICD9
ACEIs, angiotensin converting enzyme inhibitors; ARAs, aldosterone receptor antagonists; ARBs, angiotensin receptor blockers; BBs, beta-blockers; CCBs,		

calcium channel blockers; DBP, diastolic blood pressure; HTN, hypertension; MAP, mean arterial blood pressure; SBP, systolic blood pressure

Supplementary Table S3: *CYP3A5* variant frequency in UKB population compared to dbSNP.

	UK Biobank	dbSNP global	dbSNP European
*CYP3A5**3	0.913487256	0.887372	0.929965
*CYP3A5**6	0.003421796	0.005963	0.0011
*CYP3A5**7	0.002124511	0.00474	0.00024
dbSNP, single nucleotide polymorphism database			

Supplementary Table S4: Genotype distribution in the study population and the different racial subgroups; frequency and percentages (%) of the CYP3A5 variant alleles in the total population and in different racial subgroups in the UKB population.

	CYP3A5*1	CYP3A5*3		CYP3A5*6		CYP3A5*7	
	*1/*1	*1/*3	*3/*3	*1/*6	*6/*6	*1/*7	*7/*7
Total population (N=487,171)	5,546 (1.14)	65,471 (13.44)	411,359 (84.44)	1,368 (0.28)	184 (0.04)	1,039 (0.21)	103 (0.02)
Black (N=7,641)	2,080 (27.22)	1,784 (23.35)	485 (6.35)	1,124 (14.71)	158 (2.07)	884 (11.57)	95 (1.24)
White (N=459,116)	2,038 (0.44)	57,298 (12.48)	399,108 (86.93)	47 (0.01)	0 (0)	2 (0.0004)	0 (0)
East Asian (N=1,502)	123 (8.19)	595 (39.61)	781 (52.00)	0 (0)	0 (0)	0 (0)	0 (0)
South Asian (N=7,626)	550 (7.21)	2,724 (35.72)	4,347 (57.00)	0 (0)	0 (0)	0 (0)	0 (0)
Values are expressed as N (%) relative to the respective ancestral study population.							

Supplementary Table S5: Results of the subgroup analysis based on self-reported race; linear and logistic regression analysis of the *CYP3A5* variants and blood pressure rates and HTN.

Subgroup based on self-reported race	*CYP3A5* variant	Phenotype	Beta [mmHg] for blood pressure rates/ OR for HTN	2.5% CI	97.5% CI	*P*-value
White subgroup<br><br>N = 459,116	*CYP3A5**3	SBP	0.04	-0.10	0.19	0.54
		DBP	0.10	0.02	0.18	0.01*
		MAP	0.08	-0.01	0.18	0.09
		HTN	0.9996	0.98	1.02	0.97
	*CYP3A5**6	SBP	0.76	-4.31	5.83	0.77
		DBP	1.51	-1.33	4.35	0.30
		MAP	1.26	-2.10	4.60	0.46
		HTN	1.48	0.69	3.02	0.36
Black subgroup<br><br>N = 7,641	*CYP3A5**3	SBP	-0.48	-1.26	0.30	0.23
		DBP	-0.34	-0.81	0.13	0.15
		MAP	-0.39	-0.93	0.15	0.16
		HTN	1.02	0.91	1.14	0.74
	*CYP3A5**6	SBP	0.08	-0.96	1.11	0.89
		DBP	-0.07	-0.68	0.56	0.84
		MAP	-0.02	-0.73	0.69	0.96
		HTN	1.02	0.88	1.18	0.79
	*CYP3A5**7	SBP	-0.12	-1.28	1.04	0.84
		DBP	-0.03	-0.73	0.67	0.94
		MAP	-0.06	-0.86	0.74	0.89
		HTN	1.01	0.85	1.19	0.94
	*CYP3A5**3	SBP	0.74	-0.63	2.11	0.29

East Asian subgroup<br><br>N = 1,502		DBP	0.61	-0.23	1.44	0.15
		MAP	0.65	-0.31	1.61	0.18
		HTN	1.04	0.83	1.30	0.76
South Asian subgroup<br><br>N = 1,626	*CYP3A5**3	SBP	0.04	-0.58	0.66	0.90
		DBP	-0.27	-0.63	0.09	0.15
		MAP	-0.17	-0.58	0.25	0.44
		HTN	0.96	0.88	1.05	0.35
DBP, diastolic blood pressure; HTN, hypertension; MAP, mean arterial blood pressure; SBP, systolic blood pressure						
